# Sulfur Protects Pakchoi (*Brassica chinensis* L.) Seedlings against Cadmium Stress by Regulating Ascorbate-Glutathione Metabolism

**DOI:** 10.3390/ijms18081628

**Published:** 2017-07-26

**Authors:** Lili Lou, Jingquan Kang, Hongxi Pang, Qiuyu Li, Xiaoping Du, Wei Wu, Junxiu Chen, Jinyin Lv

**Affiliations:** 1 College of Life Sciences, Northwest A&F University, Yangling 712100, China; 18829784866@163.com (L.L.); jingquan_kang@163.com (J.K.); duxiaoping@aliyun.com (X.D.); wuwei9585@163.com (W.W.); chenjunxiu1995@gmail.com (J.C.); 2 College of Agronomy, Northwest A&F University, Yangling 712100, China; hxpang@163.com; 3 Innovation Experimental College, Northwest A&F University, Yangling 712100, China; 18829349240@163.com

**Keywords:** cadmium, sulfur metabolism, ascorbate-glutathione cycle, transcriptional regulation, pakchoi

## Abstract

Cadmium (Cd) pollution in food chains pose a potential health risk for humans. Sulfur (S) is a significant macronutrient that plays a significant role in the regulation of plant responses to diverse biotic and abiotic stresses. However, no information is currently available about the impact of S application on ascorbate-glutathione metabolism (ASA-GSH cycle) of Pakchoi plants under Cd stress. The two previously identified genotypes, namely, Aikangqing (a Cd-tolerant cultivar) and Qibaoqing (a Cd-sensitive cultivar), were utilized to investigate the role of S to mitigate Cd toxicity in Pakchoi plants under different Cd regimes. Results showed that Cd stress inhibited plant growth and induced oxidative stress. Exogenous application of S significantly increased the tolerance of Pakchoi seedlings suffering from Cd stress. This effect was demonstrated by increased growth parameters; stimulated activities of the antioxidant enzymes and upregulated genes involved in the ASA-GSH cycle and S assimilation; and by the enhanced ASA, GSH, phytochelatins, and nonprotein thiol production. This study shows that applying S nutrition can mitigate Cd toxicity in Pakchoi plants which has the potential in assisting the development of breeding strategies aimed at limiting Cd phytoaccumulation and decreasing Cd hazards in the food chain.

## 1. Introduction

Heavy metal contamination in the soil has become a severe environmental problem worldwide and thus has attracted considerable attention [1,2,3,4]. Among the toxic heavy metals, Cadmium (Cd) is recognized as one of the major widespread environmental pollutants and highly toxic to plants and humans, thus affecting their growth and health [5,6,7]. Cd is released primarily into the environment by anthropogenic activities, such as those involved in industries for electroplating, leather tanning, metal finishing, steel production, pigment manufacturing, and wastewater irrigation [7,8]. Previous studies reported that Cd can inhibit plant growth and cause diseases in plants by altering the several physiological processes [9,10,11].

Cadmium is a nonredox metal that cannot produce reactive oxygen species (ROS) via Fenton and Haber-Weiss reactions. However, some studies have demonstrated that oxidative stress is a major component of Cd phytotoxicity [12,13] and thus mediates cellular damage [6]. Under this condition, plants have evolved a list of complex metabolic strategies to counterbalance toxicity due to Cd stress [3]. Some of these strategies are cellular wall binding [14,15], plasma membrane pumping, restriction of uptake and transport [16,17], extra- and intracellular chelation, sequestration in vacuoles [18], antioxidant system composed of enzymatic and non-enzymatic components, and signaling mechanisms [19,20].

Ascorbate-glutathione (ASA-GSH) cycle protects the cells against ROS [21,22]. ASA participates in growth processes, electron transport, or ROS scavenging through ascorbate peroxidase (APX). ASA is oxidized to monohydroascorbate (MDHA) by ascorbic acid oxidase (AAO) and APX. MDHA is an unstable radical with a relatively short lifetime and reduced rapidly to ASA and dehydroascorbate (DHA) [23,24]. MDHA is reduced to ASA according to monodehydroascorbate reductase (MDHAR) using nicotinamide adenine dinucleotide phosphate (NADPH) as electron donor. Furthermore, DHA is reduced to ASA by dehydroascorbate reductase (DHAR). GSH is oxidized to oxidized glutathione (GSSG) when DHA is reduced to ASA. GSSG can be reconverted to GSH by receiving electron from NADPH through the activity of glutathione reductase (GR) [25,26,27]. This finding confirms the fundamental role of GSH in antioxidative defense. This pathway is also complemented by other antioxidant enzymes, such as glutathione peroxidase and glutathione-*S*-transferases (GST) [28]. Upregulating the genes encoding these enzymes enables the active ASA-GSH pathway to detoxify various ROS.

Studies have shown that appropriate application of plant nutrients may alleviate the stress-induced negative effects [29], decrease Cd concentration, and increase biomass and grain yield [30,31]. Plant nutrients help alleviate physiological stress caused by excessive Cd [32,33]. Among the various mineral nutrients involved in Cd detoxification, the role of sulfur (S) in Cd tolerance is the most significant. It plays a vital role in plant defense responses to stress [23,34,35]. S is a structural constituent of amino acids, coenzymes, proteins, and most defense compounds, including phytochelatins (PCs), GSH, glucosinolates, and vitamins, that protect plants from oxidative damage and adverse environmental stresses [36]. Cysteine is produced via a cascade of enzymatic reactions followed by a series of reactions. It is the substrate for glutathione and PC peptides through key enzymes (γ-glutamylcysteine synthetase (γ-ECS), glutathione synthetase, and PC synthase) in response to oxidative stresses, such as heavy metal exposure [37,38,39]. Furthermore, the genes of key enzymes (ATP sulfurylase, *O*-acetylserine(thiol)lyase, and γ-ECS) engaged in S assimilation in Pakchoi plants are engaged in Cd tolerance and detoxification [38,40].

Pakchoi (*Brassica chinensis* L.) plant is one of the most important leafy cruciferous vegetables widely cultivated in southern China [41,42]. Pakchoi plant possesses a high capacity for Cd accumulation in its leaves; this condition can cause serious potential health risks in people eating Pakchoi plant grown in Cd-polluted areas [43,44,45]. Therefore, reducing Cd accumulation in the edible parts of this vegetable is essential. Manipulating the enzymes involved in S assimilation, ASA-GSH cycle, and PC content may contribute to Cd detoxification. Thus, the present work is designed to provide insights into the role of S in alleviating Cd stress, and, to elucidate possible mechanisms of S-mediated physiological changes of Cd toxicity in Pakchoi seedlings. The influence of S on Cd-induced changes in two different Cd-tolerating Pakchoi seedlings are studied, particularly with respect to the following variables: growth, Cd uptake and translocation, tissue lipid peroxidation content, nonprotein thiol (NPT), PCs, ROS production, antioxidant defense enzymes, non-enzymatic antioxidants involved in ASA-GSH cycle, gene expression engaged in S metabolism and ASA-GSH cycle.

## 2. Results

### 2.1. Plant Growth Parameters

**Figure 1:**
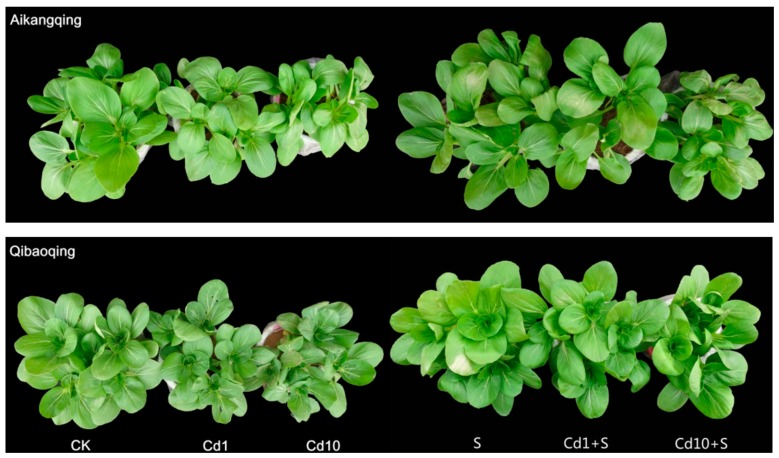
Phenotypes of 40-days-old pakchoi plants grown under different growth conditions of Cd and S. CK, control; Cd1, 1 mg·kg^−1^ Cd; Cd10, 10 mg·kg^−1^ Cd; S, 50 mg·kg^−1^ S; Cd1 + S, 1 mg·kg^−1^ Cd and 50 mg·kg^−1^ S; Cd10 + S, 10 mg·kg^−1^ Cd and 50 mg·kg^−1^ S. Three independent biological replications were performed with one treatment, and 5 Pakchoi seedlings were planted in a pot.

The effect of Cd and S on the shoot and root growth are shown in Table 1. Cd stress decreased the growth parameters of both cultivars compared with the control (Figure 1). Meanwhile, the suppression effects of Cd stress on growth characters were stronger in Qibaoqing than those in Aikangqing. The application of 10 mg·kg^−1^ Cd caused maximum reduction compared with those control. The fresh weight of the shoots and roots was reduced by 11.0, 36.8% in Aikangqing and 24.5, 17.5% in Qibaoqing due to 1 and 10 mg·kg^−1^ soil compared with control. S application markedly decreased the inhibitory effect on plant growth, resulting in the increase in plant height, root length, and fresh weight. Exogenously applied S alone also caused a slight increase in growth compared with control. The growth parameters (except for the length of the roots in Aikangqing) of the two cultivars were higher under S treatment alone than those under the other treatments. S application (50 mg·kg^−1^) favored the growth of plants and rid the toxic effects generated by Cd.

### 2.2. Cadmium Contents, Bioconcentration Factor (BCF) and TF in the Shoots and Roots of Pakchoi Cultivars Subjected to Different Treatments

Table 2 shows the effect of increasing Cd concentration in the soil. The Cd content in the Pakchoi tissues of Aikangqing and Qibaoqing increased. Exogenous S application significantly caused the reduction of Cd content in Cd-treated seedlings. S application caused a decrease of Cd content by 35.1%, 26.8% in Aikangqing and 34.5%, 27.9% in Qibaoqing of the shoots compared with the values recorded in Cd1 and Cd10-treated seedlings, respectively. In the roots, this decrease was 27.63%, 27.25% in Aikangqing and 29.02%, 20.41% in Qibaoqing compared with the values recorded in Cd1 and Cd10-treated plants.

The BCF for two cultivars reduced as Cd level increased and S application reduced the value of BCF. In addition, the roots were significantly higher than the shoots in two cultivars and the BCF value in Aikangqing were significantly higher than that in Qibaoqing. A parallel change was also observed in TF of Cd. As clearly shown from the data of TF, S application reduces Cd translocation from the root to the shoot both in Aikangqing and Qibaoqing.

### 2.3. H_2_O_2_ and MDA Contents

**Figure 2:**
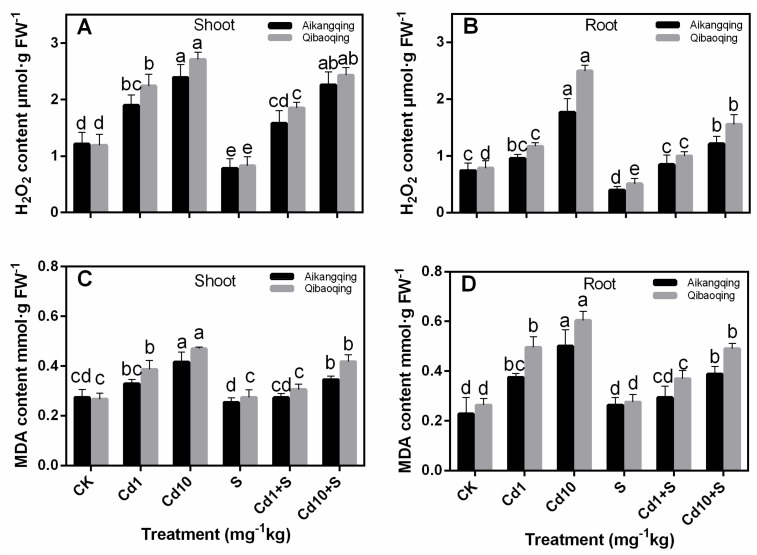
(**A**,**B**) Hydrogen peroxide (H_2_O_2_) and (**C**,**D**) malondialdehyde (MDA) contents in leaves and roots of Aikangqing and Qibaoqing. The leaves and roots of three seedlings were separately collected in one replication and three independent biological replications were performed. Different letters indicate significant differences at a *p* < 0.05 significance level.

MDA and H_2_O_2_ contents in Cd treatments were also increased with the change of Cd-treated concentration (Figure 2). However, the MDA and H_2_O_2_ contents in S application plants were considerably lower than those of Cd treatment alone. These results indicated that exogenous S application alleviated the accumulation of MDA and H_2_O_2_ induced by Cd. Additionally, the H_2_O_2_ contents of Qibaoqing were significantly higher than that in Aikangqing. Similar trends were also found in the MDA contents.

### 2.4. Enzymes of S Assimilation Pathway

**Figure 3:**
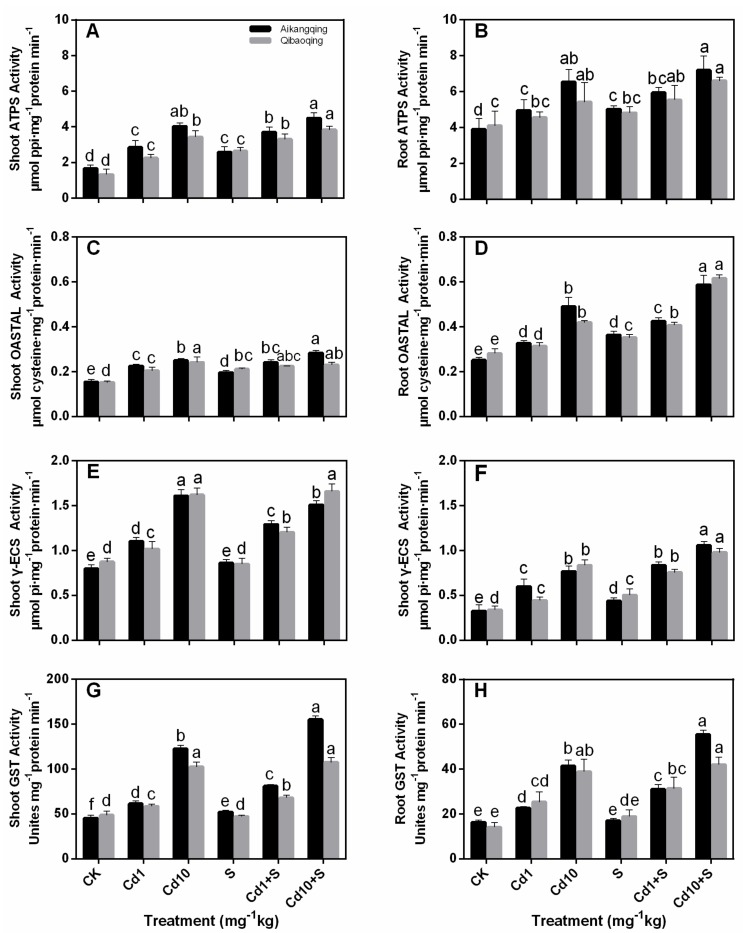
Key enzymes activities of S assimilation pathway and glutathione (GSH) metabolism. (**A**,**B**) ATP sulfurylase (ATPS); (**C**,**D**) *O*-acetylserine(thiol)lyase (OASTAL); (**E**,**F**) γ-glutamylcysteine synthetase(γ-ECS) and (**G**,**H**) GST activity in leaves and roots of Aikangqing and Qibaoqing. The leaves and roots of three seedlings were separately collected in one replication and three independent biological replications were performed. Different letters indicate significant differences at a *p* < 0.05 significance level.

ATP sulfurylase (ATPS), *O*-acetylserine(thiol)lyase (OASTAL), and γ-glutamylcysteine synthetase(γ-ECS) are all key enzymes in S assimilation pathway. They showed similar trends in the shoots and roots under S and Cd treatments. All the three enzymes improved with increasing Cd treatments. S application enhanced their activities. A considerable increase in Aikangqing was observed (Figure 3A–F).

GST activity showed an increasing trend as Cd concentration increased in the soil of both cultivars. Cd10 and S treatments increased the shoot and root GST activity. The increase was 1.97- and 2.26-fold in Aikangqing (*p* < 0.05) compared with their respective controls (Figure 3G,H). GST activity was also significantly higher in Aikangqing than that in Qibaoqing under the same Cd and S exposure, respectively (except for the root at Cd1, S, and Cd1 + S treatments).

### 2.5. Determination of the Activity of Antioxidant Enzymes in the ASA-GSH Cycle

**Figure 4:**
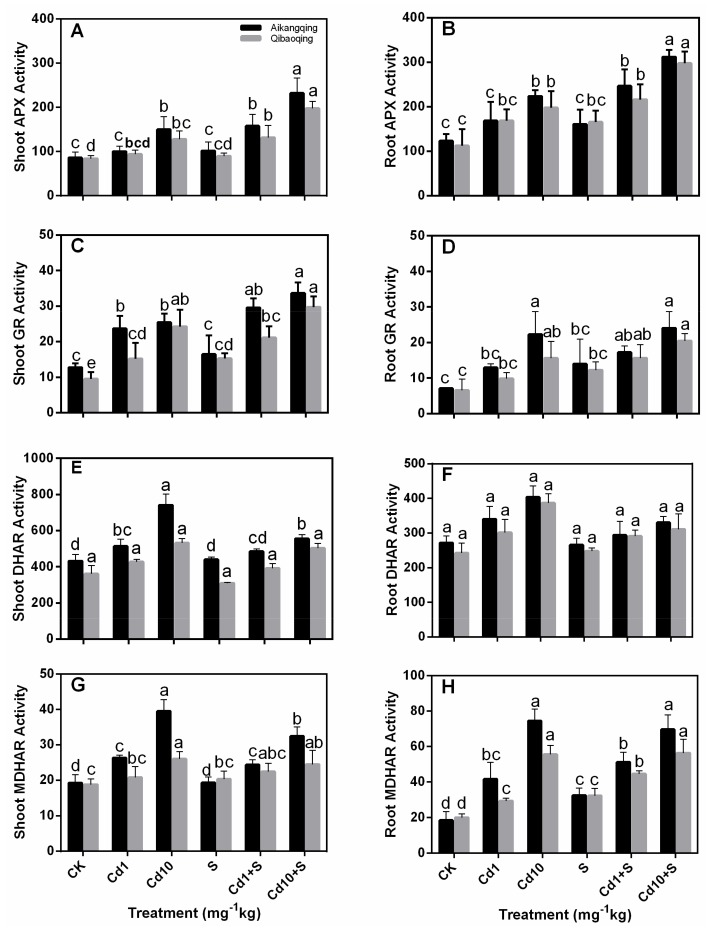
Determination of antioxidant enzymes in the Ascorbate-glutathione (AsA-GSH cycle). (**A**,**B**) ascorbate peroxidase (APX); (**C**,**D**) glutathione reductase (GR); (**E**,**F**) dehydroascorbate reductase (DHAR) and (**G**,**H**) monodehydroascorbate reductase (MDHAR) activity in leaves and roots of Aikangqing and Qibaoqing. The leaves and roots of three seedlings were separately collected in one replication and three independent biological replications were performed. Different letters indicates significant differences (*p* < 0.05). Units of these enzyme activity, U·mg^−1^protein·min^−1^.

The four key enzymes that clean ROS via maintaining a highly reduced contents of GSH and ASA, namely, APX, GR, DHAR, and MDHAR, showed similar responses to different treatments in both tissues of the Aikangqing and Qibaoqing cultivars (except DHAR activity) (Figure 4).

The activities of APX, GR, MDHAR, and DHAR were significantly enhanced by Cd1 and Cd10 treatments. Meanwhile, the APX, GR, MDHAR increased with S application. DHAR showed differential response to S under Cd exposure in contrast to APX, GR, and MDHAR, DHAR activity was reduced compared with control.

### 2.6. Determination of Glutathione, Ascorbate, PCs, and NPT

**Figure 5:**
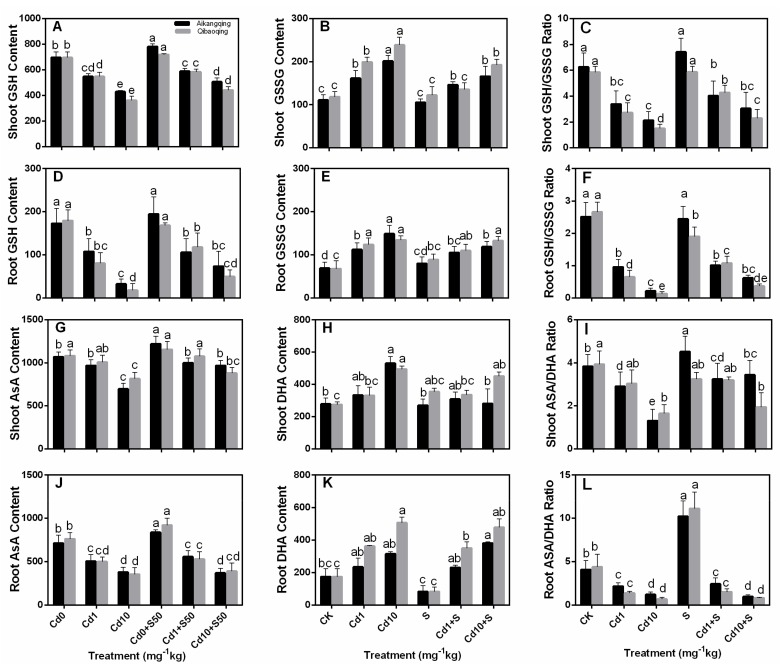
Effects of exogenous S on content of (**A**,**D**) glutathione (GSH); (**B**,**E**) oxidized glutathione (GSSG); (**G**,**J**) ascorbate (ASA); (**H**,**K**) dehydroascorbate (DHA); (**C**,**F**) GSH/GSSG ratio, and (**I**,**L**) ASA/DHA ratio in roots and leaves of Pakchoi seedlings suffered from Cd stress. The leaves and roots of three seedlings were separately collected in one replication and three independent biological replications were performed. Different letters indicates significant differences (*p* < 0.05). Units of GSH, GSSG, ASA and DHA content, nmol·g·FW^−1^.

Different cadmium concentrations triggered a significant decrease in GSH and ASA content in the shoots and roots of both cultivars compared with control seedlings (Figure 5A,B,G,H). Exogenous S improved ASA and GSH contents, and declined GSSG and DHA contents in Cd1, as well as in Cd10-treated seedlings (Figure 5D,E,J,K). The ratio of GSH relative to GSSG was higher in Aikangqing than that of Qibaoqing. Cd exposure resulted in a lower ratio of GSH relative to GSSG in both cultivars, but the GSH to GSSG ratio was higherer in the S application plants. Similar tendencies were also found in the ratio of ASA relative to DHA.

**Figure 6:**
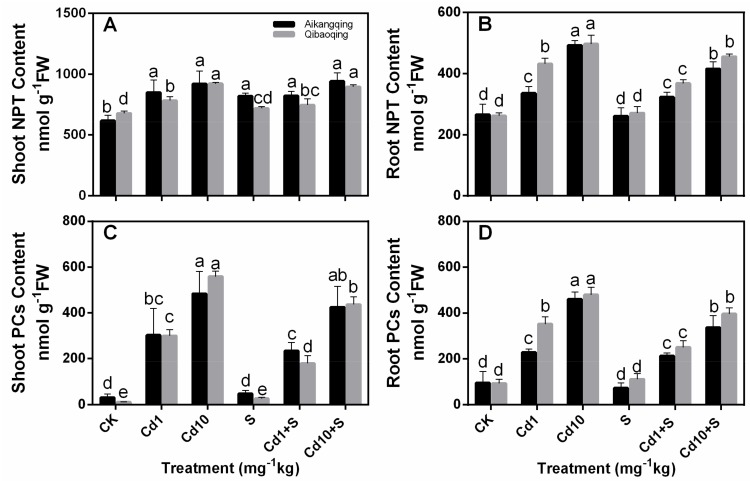
Effects of S addition on (**A**,**B**), nonprotein thiol (NPT) and (**C**,**D**), phytochelatins (PCs) contents in both tissues of the Pakchoi cultivars Aikangqing and Qibaoqing under Cd stress. Data show the mean ± SD of three replicates. Different letters indicate significant differences at a *p* < 0.05 significance level.

The NPT content in the roots significantly improved in both cultivars under different Cd concentration stresses compared with control plants (Figure 6A,B). No significant differences were observed in the shoots. However, the NPT content decreased in the shoots and roots of two cultivars because of interactive S and Cd treatments compared with solely Cd-stressed plants.

PCs content drastically increased in both tissues and cultivars treated with Cd1 and Cd10 (Figure 6C,D). S application resulted in reduction of PCs content in both the cultivars compared with Cd-stressed seedlings. S application increased PCs content by 55.8% and 128% of the shoots in Aikangqing and Qibaoqing respectively compared with the control plants in the absence of Cd treatment. However, PCs content reduced by 23.1% and increased by 20.8% in the roots of Aikangqing and Qibaoqing respectively compared with control (CK).

### 2.7. Transcript Levels of Gene-Encoding Enzymes Involved in S Assimilation Pathway in the Shoots and Roots of Pakchoi Seedlings

**Figure 7:**
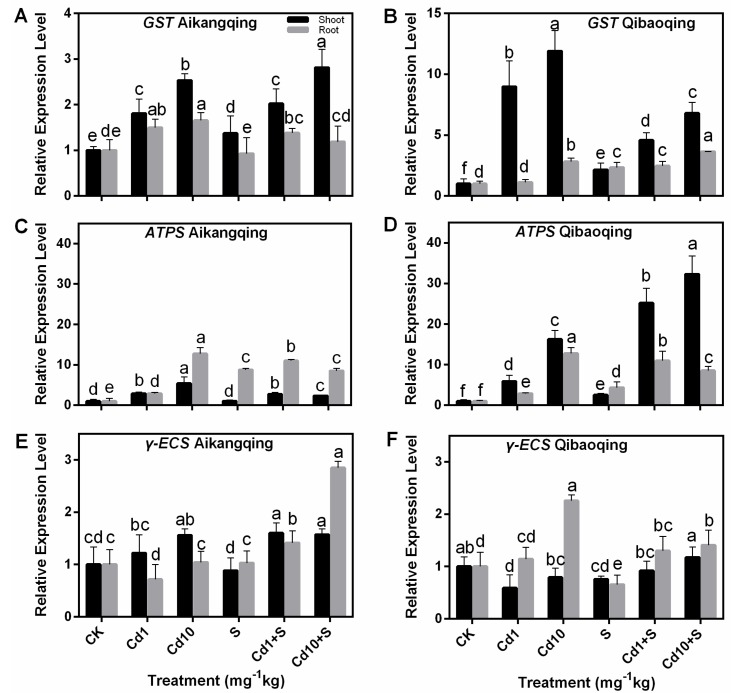
Expression of key enzymes activities of S assimilation pathway and GSH metabolism. Transcripts were analyzed by qPCR using *Actin* gene as internal control. (**A**,**C**,**E**) transcript levels of *GST, ATPS* and *γ-ECS* genes in Aikangqing; (**B**,**D**,**F**) transcript levels of the three genes in Qibaoqing, respectively. The leaves and roots of three seedlings were collected in one replication and three independent biological replications were performed. Each value is the mean ± standard deviation of three independent measurements. Different letters indicate significant differences (*p* < 0.05).

*GST* expression in the Aikangqing shoots and roots was upregulated by Cd treatment. S treatment enhanced expression of *GST* in the shoots. In the roots, *GST* expression was downregulated compared with Cd treatment (Figure 7A). Furthermore, the transcription of the *GST* gene increased sharply at Cd stress in the shoots in Qibaoqing, whereas it increased gradually in the roots. Exogenous S decreased the transcript levels of the *GST* gene in the shoots, whereas it was enhanced in the roots (Figure 7B).

*ATPS* expression in the shoots and roots of both cultivars quickly increased at different levels of Cd treatment (Figure 7C,D). S and Cd treatment enhanced *ATPS* expression level, especially in the roots of Aikangqing (2.79-fold) and shoots of Qibaoqing (3.52-fold) compared with the Cd1 treatment. A similar pattern was noted with *γ-ECS* gene (Figure 7E,F). However, *γ-ECS* expression was much stable than *ATPS.* The expression levels of *γ-ECS* slowly increased in both tissues and cultivars of plants exposed to Cd and Cd + S stress (except in the shoots of Qibaoqing at Cd10 treatment).

### 2.8. Transcript Levels of Gene-Encoding Enzymes Involved in ASA-GSH Cycle

**Figure 8:**
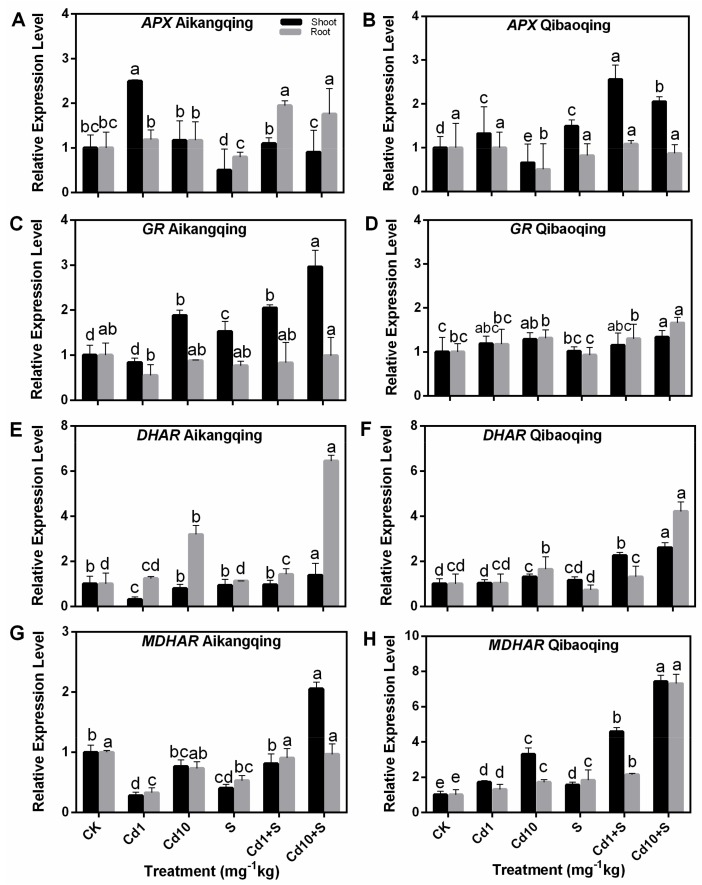
Effects of exogenous S on transcript levels of the four genes encoding ASA-GSH cycle enzymes in Pakchoi seedlings suffering from Cd stress. Transcripts were analyzed by qPCR using *Actin* gene as internal control. (**A**,**C**,**E**,**G**) transcript levels of *APX*, *GR*, *DHAR* and *MDHAR* genes in Aikangqing; (**B**,**D**,**F**,**H**) transcript levels of the four genes in Qibaoqing, respectively. The leaves and roots of three seedlings were collected in one replication and three independent biological replications were performed. Each value is the mean ± standard deviation of three independent measurements. Different letters indicate significant differences (*p* < 0.05).

Figure 8 shows that the transcription level of enzymes in ASA-GSH pathway including *APX*, *GR*, *DHAR*, and *MDHAR* increased under Cd stress. The results showed that S application increased the transcript levels of the four genes in stressed plants. However, their expression patterns were different to some extent (Figure 8). *APX* expression was markedly induced at Cd1 treatment, and then decreased at Cd10 treatment in both tissues and cultivars. Meanwhile, *APX* expression in Cd + S-treated plants was higher than those only exposed to Cd (Figure 8A,B). *GR* expression in the roots of Aikangqing remained almost constant but was significantly enhanced in a dose-dependent manner in the shoots. The transcript levels of *GR* in both tissues of Qibaoqing gradually increased with the increased dose of Cd (Figure 8C,D). Different to the *APX* expression, *DHAR* expression was significantly induced at Cd10 treatment in both tissues and cultivars, and it was enhanced in Cd + S treatment (Figure 8E,F). A parallel change was also observed in *MDHAR* gene (Figure 8G,H).

## 3. Discussion

Cadmium (Cd) has been known as a highly ecotoxic heavy metal and environmental hazard that causes detrimental effects in plant growth and human health [7,46,47,48,49]. Alleviation of metal toxicity by sulfur (S) application has been observed in many plant species in previous studies [34,50,51]. Moreover, all the results in these studies demonstrated that S addition can enhance plant growth under metal toxicity. The results of the current study supported these findings. Our results showed that exposing two cultivar Pakchoi seedlings to Cd stress can considerably hamper plant growth in dose dependent manner and sulfur restored the growth (Figure 1, Table 1). The growth inhibition caused by Cd stress might be due to Cd-induced generation of ROS that involved various factors, such as H_2_O_2_, superoxide radical, and hydroxyl radical promoting the MDA contents through lipid peroxidation [11,52,53]. The alleviative effects may result from S involving multiple detoxification mechanisms [54]. The current work supports the hypothesis that sufficient S may enable the chelation of more Cd in plant roots, subsequently limiting its translocation from root to shoot [55]. We found that Cd accumulation was greater in the roots, and less was observed in the shoots (Table 2). BCF is a reliable indicator of metal accumulation capacity of plants [56,57]. According to Odjegba and Fasidi [58] and Zayed et al. [59], “a good metal accumulating plant should have a >1000 BCF value.” The current study shows that BCF value of both the shoots and roots in Aikangqing was higher than 1000, and therefore can be considered to be metal accumulators. S application reduces Cd translocation to the shoot in both Aikangqing and Qibaoqing as evident from Cd concentration, TF, and BCF. Thus, improving S supply might enhance the plant tolerance against Cd by reducing the translocation from the root to the shoot.

Oxidative stress induced by over production of ROS is a common toxic effect shared by diverse biotic and abiotic stresses [60,61,62,63]. The ROS increase may lead to lipid peroxidation indicated by MDA level. This process was also associated to the formation of H_2_O_2_ [64,65]. Therefore, H_2_O_2_ and MDA contents can suggest the level of oxidative stress in plants [66]. Cd stress induced a significant damage effect on Pakchoi plant suggested by the enhanced H_2_O_2_ and lipid peroxidation in both tissues of Aikangqing and Qibaoqing in the current work. However, S application to Cd-stressed seedlings significantly alleviated Cd-generated oxidative damage (Figure 2). ROS level in plant cells is strictly regulated by an antioxidant system, which comprises enzymatic and non-enzymatic antioxidants [3,65]. ASA and GSH are molecules of major non-enzymatic antioxidants. In addition, the enzymes and antioxidants in “the ASA-GSH pathway play a vital role in preserving the balance between ROS production and scavenging in plants challenged to various abiotic stress conditions” according to [27,60,67]. Previous studies demonstrated that stressful conditions can stimulate the antioxidant defense system in different plant species [27,68,69]. Most of the antioxidant enzymes in the ASA-GSH pathway (APX, GR, DHAR, and MDHAR) showed significant increases in both tissues of plants under Cd stress in our study. This result indicates that Pakchoi plant has activated antioxidant defense-system response to Cd stress. Moreover, the antioxidant enzyme activities in Aikangqing were higher than those in Qibaoqing, which suggested that the role of S application in alleviating abiotic stresses is closely related to the efficiently regulated antioxidant defense system [23,34,70]. Application of exogenous S increased the activities of antioxidant enzymes in the ASA-GSH cycle (APX, GR, DHAR, and MDHAR) and key enzymes of S assimilation pathway (ATPS, γ-ECS, OASTAL, and GST) caused by Cd stress as revealed by increased growth parameters (plant height, root length, and fresh weights), and diminished H_2_O_2_ and MDA content in this study (Table 1, Figure 2). These findings suggest that S application enhances the tolerance of Pakchoi plant to Cd stress similar to the previous studies in maize [71], rice (*Oryza sativa* L.) [72], garden cress (*Lepidium sativum* L.) [47], and yellow mustard (*Sinapis alba* L.) [73]. This phenomenon may be linked with S application inducing Cd decrease in plant responsible for ROS production.

A negative correlation existed between the Cd stress and major non-enzymatic antioxidants (ASA and GSH) in Cd-stressed plants, and applying S increased the ASA and GSH contents slightly (Figure 5) similar to the previous studies in mung bean and maize [74,75]. GSH is a precursor of PCs, which plays an important role in controlling cellular heavy metal concentration [76,77,78]. The depletion of GSH pools can be attributed to the demand in PCs synthesis, one of the most vital components of NPT. However, the thiol pool responses can be partially reversed by supplying S. S application probably regulates GSH content by increasing its biosynthesis. This effect can be seen from the upregulation of γ-ECS and ATPS activity and transcript levels (Figure 3 and Figure 7). NPT primarily constitute GSH and PCs. NPT content was enhanced under Cd stress and S supply suggesting its importance in Cd detoxification. S assimilation regulates NPT biosynthesis. The results from our studies indicated that S application can markedly increase the capabilities of counteracting the impact of oxidative damage triggered by Cd toxicity in Pakchoi plants.

In response to Cd stress, the plant may regulate the expression levels of genes engaged in S assimilation and ASA-GSH cycle providing an increased flux through the complete pathway when S is not limiting [79]. Overexpression of S-assimilating enzymes enhances tolerance to heavy metals [38,40]. The expression levels of seven genes encoding key enzymes involved in S assimilation (*ATPS*, *γ-ECS*, and *GST*) and ASA-GSH pathway enzymes (*GR*, *APX*, *DHAR*, and *MDHAR*) were determined in Cd-stressed Pakchoi seedlings to identify their associations with the Cd stress and S application in our work. The contemporary work showed that plants activate the S-assimilation pathway by increasing the transcription of related genes. This increase can provide a great supply of Cys or GSH for PC biosynthesis under cadmium stress. The transcript profiles of these genes varied in a dose-dependent manner. Most of the transcript levels of the genes were markedly enhanced by S application at different levels of cadmium treatment (Figure 7 and Figure 8). These results are also supported by several previous studies on Indian mustard [80], tobacco [81,82], *Brassica rapa* [83], *Arabidopsis* [40,84], and poplar [38].

**Figure 9:**
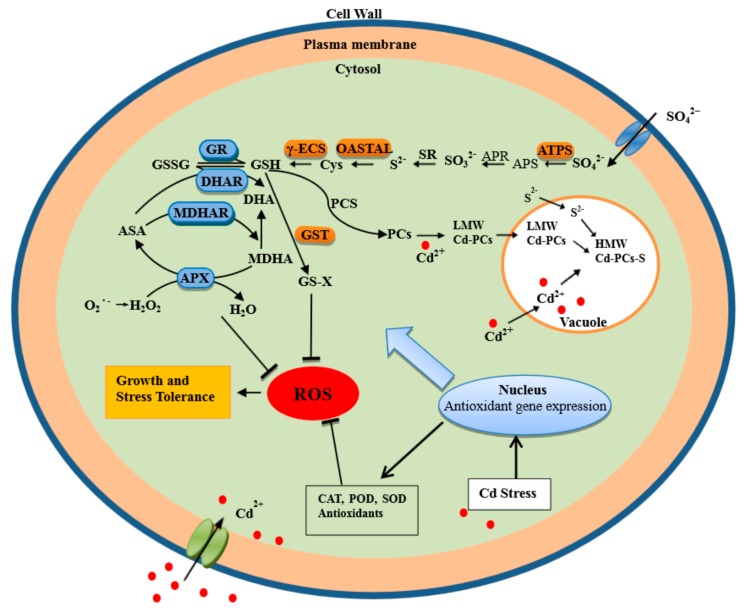
Simplified metabolic scheme and signal transduction pathway for the role of sulfur in regulating Cd accumulation and tolerance.

S uptake results in the initial formation of the first steady product cysteine via a set of enzymatic reactionsare summarized in Figure 9. Cysteine formation results in GSH synthesis, a non-enzymatic antioxidant acknowledged engaged in ASA-GSH cycle to regulate Cd detoxification either via quenching ROS or biosynthesizing PCs and NPT that combines Cd and separates it into vacuole. Regulation of key enzymes in S assimilation, ASA-GSH cycle or PCs content might result in Cd detoxification. However, more studies are needed to obtain more insights into the mechanisms of regulation of S assimilation involved in Cd toxicity in Pakchoi plants at molecular level. Evidence on signaling molecules and mechanisms still remains to be clarified.

## 4. Materials and Methods

### 4.1. Plant Cultivation

A pot culture experiment was conducted from June to October 2015 under open field conditions in the Northwest A&F University, Yangling, Shaanxi, China (34°22′ N, 108°26′ E, 526 m elevation). Surface soil (0–30 cm) was collected from the local agricultural farm (earth-cumuli-orthic anthrosols). Surface soil was passed through a 5 mm sieve. Then, 2.5 kg soil samples were filled with each plastic pot (20 cm in diameter and 15 cm in height) after being artificially mixed with Cd (in CdCl_2_·2.5H_2_O solution) and S (in Na_2_SO_4_ solution). Since Na_2_SO_4_ is the only source of sulfur, the amount of NaCl was simultaneously increased to restore standard sodium concentration in sulfate-deprived treatment. Six treatments including the control (without addition of Cd and S) were arranged and applied according to the following treatments:

(1) Control (CK), 0 mg·kg^−1^ CdCl_2_·2.5H_2_O + 0 mg·kg^−1^ Na_2_SO_4_;
(2) Cd1, 1 mg·kg^−1^ CdCl_2_·2.5H_2_O + 0 mg·kg^−1^ Na_2_SO_4_;
(3) Cd10, 10 mg·kg^−1^ CdCl_2_·2.5H_2_O + 0 mg·kg^−1^ Na_2_SO_4_;
(4) S, 0 mg·kg^−1^ CdCl_2_·2.5H_2_O + 50 mg·kg^−1^ Na_2_SO_4_;
(5) Cd1 + S, 1 mg·kg^−1^ CdCl_2_·2.5H_2_O + 50 mg·kg^−1^ Na_2_SO_4_;
(6) Cd10 + S, 10 mg·kg^−1^ CdCl_2_·2.5H_2_O + 50 mg·kg^−1^ Na_2_SO_4_.

The soil subsamples were fully watered and then left for equilibration outdoors for approximately 60 days.

After 60 days, two identified Pakchoi genotypes, namely, Aikangqing (a Cd-tolerant cultivar) and Qibaoqing (a Cd-sensitive cultivar) were used in the present study. In each pot, 10 seeds were sown directly into the soil, and the number of seedlings were reduced to 5 per pot after two weeks. Four replicates were used for each treatment. The plants were grown for 40 days, and then harvested for analysis.

### 4.2. Measurement of Morphological Features

Five plants from four separate pots of each treatment were used as biological replicates after 40 days of growth. The roots and shoots with same sizes were harvested and rinsed with tap water to remove soil. The roots were dipped in 10 mM ethylenediaminetetraacetic acid (EDTA) for 10 min, and then rinsed thoroughly with distilled water. The plants into roots and shoots were separated. Fresh shoot and root samples were frozen in liquid nitrogen (N_2_) and stored at −80 °C until further physiological and biochemical analyses.

Morphological growth parameters, such as root fresh weight, root length, root dry weight, shoot fresh weight, shoot height, and shoot dry weight were measured.

The roots and shoots of the plants were separated manually, and then dried at 80 °C in an oven to a constant weight before obtaining the dry weight. The dried plant tissues were prepared for Cd measurement.

The collected soil samples were dried and sieved through a 2 mm sieve.

### 4.3. Cd Determination by Atomic Absorption Spectroscopy

The dried shoots, roots, and soil samples were ground. The resulting powder (approximately 0.2 g) was used for digestion with a 10 mL solution of HNO_3_ and HClO_4_ (*v*/*v* = 4:1) at 220 °C. Cd concentration was measured through flame atomic absorption spectroscopy (Hitachi 180-80, Japan).

### 4.4. Bioconcentration and Translocation Factors

The bioconcentration factor (BCF) is defined as an excellent index of metal accumulation capacitance in plants. The translocation factor (TF) can be used to evaluate a plant’s potential for phytoremediation purpose. The BCF and TF were calculated following methods of Shi and Cai [56], as follows:

BCF = Cd_shoot or root_/Cd_soil_, (1)

TF = Cd_shoot_/Cd_root_, (2)

where Cd_soil_ is the concentration of total Cd in soil; Cd_shoot_ and Cd_root_ are the concentration of Cd in the shoot and root parts, respectively.

### 4.5. Measurement of Malondialdehyde (MDA) and Hydrogen Peroxide (H_2_O_2_) Content

MDA content was measured by using 2-thiobarbituric acid reactions, and the results were analyzed spectrophotometrically at 450, 532, and 600 nm as described by Hodges et al. [85]. The H_2_O_2_ content was analyzed as described by Alexieva et al. [86].

### 4.6. Activity of Enzymes of S Assimilation Pathway and ASA-GSH Cycle Metabolism

The method of Masato [87] with some modifications was used to determine the reduced ASA and oxidized DHA ascorbate contents. Total GSH and GSSG were measured following the method of Anderson [88].

Total NPT were measured following the method of Ellman [89]. PC content was calculated according to the method of Duan et al. [90]. The latter method subtracts the GSH content from that of the NPT (PCs = NPT − total GSH).

APX activity (EC 1.11.1.11) was measured by monitoring the change in absorbance at 290 nm, and GR (EC 1.6.4.2) was monitored at 340 nm following the method of Nakano and Asada [91] and Smith et al. [92].

Up to 0.5 g plant samples were extracted in 5 mL medium containing 50 mM phosphate buffer, pH 7.5, 1 mM EDTA, and 1 mM dithiothreitol to estimate GST (EC 2.5.1.18) activity. The enzyme activity was assayed in a reaction mixture containing 100 mM potassium phosphate buffer (pH 6.5), 1 mM EDTA, 1 mM 1-chloro-2, 4-dinitrobenzene, 1 mM GSH, and the enzyme extract. The increase in absorbance was determined at 340 nm. The activity was calculated using an extinction coefficient of 9.6 mM·cm^−1^ expressed as U·min^−1^ mg·protein^−1^ [93].

ATP sulfurylase (ATPS, EC 2.7.7.4) activity was assayed using molybdate-dependent formation of pyrophosphate as described by the method of Lappartient and Touraine [94]. *O*-acetylserine(thiol)lyase (EC 4.2.99.8) was measured according to the method by Riemenschneider et al. [95]. Assays for γ-ECS (EC 6.3.2.2) activities and the method according to Seelig and Meister (1984) were performed with slight modifications. Enzyme activity was presented as μmol Pi/min mg protein.

DHAR (EC 1.8.5.1) activity was measured at 265 nm following the method of Pinto et al. [96]. In this method, the GSH-dependent production of ASA is determined. The enzyme extract was added to a reaction mixture containing 50 mM phosphate buffer (pH 7.0), 20 mM GSH, and 2 mM DHA. The reaction was initiated by adding DHA. One unit of DHAR activity was defined as a 0.01 increase in absorbance at 265 nm for 1 min.

MDHAR (EC 1.6.5.4) activity was determined following the method of (Pinto,TommasiandGara [96]) with slight modifications. The reaction mixture contained 50 mM phosphate buffer (pH 7.0), 2 mM ASA, 0.25 U AAO, 2 mM NADPH, and enzyme extract. The reaction was initiated by adding AAO. One unit of MDHAR activity was defined as a 0.01 increase in absorbance at 290 nm for 1 min.

### 4.7. RNA Extraction and Quantitative Real-Time Polymerase Chain Reaction

Total RNAs of the shoots and roots were extracted from the samples of the three independent biological replicates for each of genotypes using the TRIzol reagent (Invitrogen) according to the manufacturer’s instructions. The first strand of cDNA was synthesized using 1.0 μg total RNA, 0.5 μg oligo d (T) 18 primer, and reverse transcriptase system (DRR037A; Takara, Dalian, China) at a total volume of 20 μL. The primers used for qPCR were designed according to the corresponding sequence on Primer 6, and the primers are listed in Table S1. Amplification through quantitative real-time polymerase chain reaction was performed with a Thermal Cycler Dice Real Time System III (Takarabio Inc., Otsu, Shiga, Japan) using an RT-PCR master mix (DRR820A; Takara) according to the user manual. The expression levels of the target genes were normalized using actin as an endogenous control. All reactions were run in triplicate. The quantification of gene expression levels relative to the control was determined by 2^−ΔΔ*C*t^ method.

### 4.8. Statistical Analysis

All the results presented were the mean values of three replications. All data were expressed as means ± standard deviation (SD). The one-way analysis of variance was performed by SPSS software version 19 (SPSS Inc., Chicago, IL, USA). Significant differences from the control values were determined at *p* < 0.05.

## 5. Conclusions

In this current study, morpho-physiological findings support that sulfur plays a critical role to withstand Cd toxicity and to restore the normal growth and development in Pakchoi plants. Decreased Cd uptake and translocation, as well as improved compartmentation, readjustment of redox homeostasis, and strengthened antioxidant capacity by further upregulating key enzyme genes as indicated by elevated ascorbate levels contribute to the S effect. These findings showed that Pakchoi plants adjust their response to excess Cd by S nutrient supplementation, including the elaborated regulation of Cd distribution and ROS elimination. Magnitude of Cd induced toxicity was low in Aikangqing, which correlated strongly with effective ASA-GSH cycle and strong defense response. S as a nutrient element can be used in agricultural production to reduce Cd toxicity and increase sustainability. These results may stimulate further studies via enhancing soil fertility and ameliorating the contaminated soil to achieve breeding of low Cd accumulating cultivars for Pakchoi plants.

## Figures and Tables

**Table 1:** Effect of exogenous S (50 mg/kg) application on growth characteristics of Pakchoi seedlings under different concentrations of Cd stress for 40 days.

Growth Parameters	CK	Cd1	Cd10	S	Cd1 + S	Cd10 + S
Aikangqing						
Shoot fresh weight (g·plant^−^^1^)	39.59 ± 4.43 ^b^	35.25 ± 3.61 ^bc^	25.66 ± 2.84 ^c^	52.66 ± 12.21 ^a^	49.91 ± 7.89 ^a^	38.42 ± 8.24 ^bc^
Shoot height (cm)	23.1 ± 1.18 ^ab^	20.67 ± 3.46 ^cd^	18.70 ± 2.09 ^d^	24.07 ± 0.81 ^a^	21.50 ± 0.44 ^bc^	20.53 ± 1.08 ^cd^
Root fresh weight (g·plant^−^^1^)	3.18 ± 0.59 ^b^	2.01 ± 0.38 ^cd^	1.52 ± 0.37 ^d^	4.18 ± 0.2 ^a^	2.35 ± 0.29 ^c^	1.62 ± 0.47 ^d^
Root long (cm)	33.25 ± 0.49 ^a^	29.68 ± 3.06 ^ab^	24.12 ± 4.42 ^c^	30.94 ± 4.95 ^ab^	27.43 ± 2.51 ^ab^	24.62 ± 4.23 ^bc^
Qibaoqing						
Shoot fresh weight(g·plant^−^^1^)	46.05 ± 4.63 ^ab^	34.78 ± 5.30 ^cd^	26.83 ± 3.89 ^d^	47.70 ± 5.35 ^a^	37.43 ± 4.51 ^bc^	33.41 ± 4.37 ^cd^
Shoot height (cm)	19.73 ± 0.51 ^ab^	17.30 ± 1.44 ^bc^	15.63 ± 1.24 ^c^	20.97 ± 2.52 ^a^	17.00 ± 0.75 ^bc^	16.48 ± 1.03 ^c^
Root fresh weight (g·plant^−^^1^)	2.40 ± 0.46 ^b^	1.98 ± 0.43 ^bc^	1.77 ± 0.27 ^c^	3.00 ± 0.43 ^a^	2.23 ± 0.38 ^b^	2.01 ± 0.31 ^bc^
Root long (cm)	28.58 ± 3.57 ^ab^	22.46 ± 1.95 ^cd^	21.08 ± 2.89 ^d^	29.68 ± 2.66 ^a^	27.46 ± 1.19 ^ab^	25.78 ± 2.84 ^bc^

Data are mean ± SD (*n* = 5). Different letters indicate significant difference at *p* < 0.05.

**Table 2:** Cadmium contents (mg·kg^−1^ dry weight), BCF (%), and TF (%) in the shoots and roots of Pakchoi cultivars subjected to different treatments.

Cultivars	Treatments	Cd Content (mg·kg^−1^ DW)		BCF (%)		TF (%)
		Shoots	Roots	Shoots	Roots	
Aikangqing	Cd1	11.83 ± 2.09 ^b^	16.14 ± 2.52 ^c^	1214	1656	73
	Cd10	45.03 ± 4.23 ^a^	69.92 ± 1.67 ^a^	521	756	69
	Cd1 + S	7.68 ± 0.39 ^b^	11.68 ± 0.23 ^d^	772	1175	66
	Cd10 + S	32.97 ± 2.71 ^a^	50.86 ± 3.95 ^b^	352	543	65
Qibaoqing	Cd1	9.53 ± 1.72 ^c^	15.16 ± 0.22 ^c^	971	1544	63
	Cd10	34.56 ± 2.59 ^a^	58.19 ± 2.65 ^a^	422	711	59
	Cd1 + S	6.24 ± 0.84 ^c^	10.76 ± 0.78 ^d^	647	1116	58
	Cd10 + S	24.91 ± 3.28 ^b^	46.31 ± 1.99 ^b^	268	499	54

The Cd content of Pakchoi seedlings is uniform, which is zero, because Cd is not integrated in soil in CK treatment. Different letters in each column indicate significant differences between mean ± SD of treatments (*n* = 3) at a *p* < 0.05 significance level.

## Supplementary Materials

Supplementary materials can be found at www.mdpi.com/1422-0067/18/8/1628/s1.

## Abbreviations

Cd	cadmium
S	sulfur
ASA	ascorbate
GSH	glutathione
PCs	phytochelatins
NPT	nonprotein thiol
ROS	reactive oxygen species
APX	ascorbate peroxidase
GR	glutathione reductase
DHAR	dehydroascorbate reductase
MDHAR	monodehydroascorbate reductase
GSSG	oxidized glutathione
DHA	dehydroascorbate
GST	glutathione-*S*-transferases
ATPS	ATP sulfurylase
OASTAL	*O*-acetylserine(thiol)lyase
γ-ECS	γ-glutamylcysteine synthetase
H_2_O_2_	hydrogen peroxide
MDA	malondialdehyde
BCF	bioconcentration factor
TF	translocation factor
