# Supplementary material for: Sulfur Protects Pakchoi (Brassica chinensis L.) Seedlings against Cadmium Stress by Regulating Ascorbate-Glutathione Metabolism
Source: Int J Mol Sci. 2017 Jul 26;18(8):1628. doi: 10.3390/ijms18081628 (PMC5578019; doi:10.3390/ijms18081628)
Supplement: Supplementary file 1 [file ijms-18-01628-s001.pdf]

Table S1. DNA sequences of PCR primers were used for qPCR determination of the seven ASA-GSH biosynthesis-related and sulfur metabolism genes in Pakchoi seedlings.

Genes	Accession no.	Primer pairs
APX	**AB901369.1**	**F: TCCCTTCGGAACAATGAG**
		**R: ACAGCCACAACACCAGCA**
GR	**AF008441.2**	**F: ATTCACGCACTAACATACCT**
		**R: TCTTCGCTGAGACCCAC**
DHAR	**AB125638.1**	**F: TTTCAGCAGCAGACTTATCCT**
		**R: CAACCAGCAATCACATCCT**
MDHAR	**AF109695.1**	**F: TTTCAGCAGCAGACTTATCCT**
		**F: CAACCAGCAATCACATCCT**
GST	**JX110775.1**	**F: TCGCTCTCCACGAGAAAGAT**
		**R: CAGGCGAGAGAAGTTGGTTC**
ATPS	**U68218.1**	**F: TCGGAGGGTTCATGAGAGAG**
		**R: GATCTTCCTTGGGATGCTTG**
γ-ECS	**X95563.1**	**F: GTTTCGTGCTGGTCTTGC**
		**R: GCGGTCCTTGTCAGTGTCT**
Actin	**KU851921**	**F: GAGACTTTCAATGCCCCTGC**
		**R: CCATCTCCAGAGTCGAGCACA**
